# Accuracy of Three-Dimensionally Printed, Patient-Specific Drill Guides for Implant Placement in Canine Cervical Vertebrae: A Cadaveric Study

**DOI:** 10.3390/vetsci12121190

**Published:** 2025-12-12

**Authors:** Christopher J. Ponticello, Christopher L. Mariani, Joshua M. R. Carrillo, Joshua A. Zlotnick, Kristen Malinak Blodgett, Ashley Gavitt, Ola Harrysson

**Affiliations:** 1 Comparative Neuroimmunology & Neuro-Oncology Laboratory, College of Veterinary Medicine, North Carolina State University, Raleigh, NC 27607, USA; cponticdvm@gmail.com (C.J.P.);; 2 Department of Small Animal Clinical Sciences, College of Veterinary Medicine, North Carolina State University, Raleigh, NC 27607, USA; 3 Edward P. Fitts Department of Industrial and Systems Engineering, North Carolina State University, Raleigh, NC 27606, USA; oaharrys@ncsu.edu; 4 Center for Additive Manufacturing and Logistics, North Carolina State University, Raleigh, NC 27606, USA

**Keywords:** spinal surgery, neurosurgery, additive manufacturing, spinal stabilization, dog

## Abstract

The objective of this study was to assess the accuracy of autoclavable, three-dimensionally (3D) printed drill guides (3DPG) created with a rapid printing protocol and utilized by both an experienced and inexperienced surgeon in constraining the trajectory of drill tracts to intended corridors within canine cervical vertebrae. The 3DPG were designed using computed tomographic (CT) images of six complete cadaveric canine cervical vertebral columns and used to create drill tracts in all vertebrae by both an experienced and a novice surgeon. Repeat CT imaging was used to assess the entry point (EPD) and angular deviation (AD) of the actual versus planned drill tracts. Unintended cortical bone violations were also assessed. A total of 80 3DPG were used to create 144 drill tracts in 42 vertebrae. The overall mean EPD was 1.1 mm (median 0.9 mm, range 0.1–5.1 mm) and the overall mean AD was 7.3° (median 5.2°, range 0.5–33.8°). There were no differences in overall EPD (*p* = 0.85) or AD (*p* = 0.20) between the inexperienced and novice surgeons. There were no unintended cortical bone breaches. These 3DPG allowed the creation of safe and accurate drill tracts within the canine cervical spine by both an experienced and an inexperienced surgeon.

## Simple Summary

Placing surgical implants into the canine spine is indicated for a number of conditions but is technically challenging and fraught with potential danger, including damage to the spinal cord and large blood vessels. To address this problem, we created three-dimensionally printed drill guides (3DPG) that matched the surface of the vertebrae and constrained drill trajectories to intended paths. Implant corridors were drilled by both an experienced surgeon and a novice surgeon into cervical (neck) vertebrae from canine cadavers. These corridors were compared with the intended corridors using computer software. We found that the drill corridors created had minimal deviations from those intended, with no violations of the spinal cord or blood vessels. There was no difference between corridors created by the two surgeons. Thus, 3DPG may improve the safety and ease of spinal implant surgery in dogs.

## 1. Introduction

Surgical stabilization of the cervical spine is often indicated in dogs with cervical spondylomyelopathy, atlantoaxial subluxation, and vertebral fractures or luxations and is most commonly performed via a free-hand ventral approach, using metal pins or screws and polymethylmethacrylate [1,2,3]. Engaging more than one bony cortex when placing metal implants is considered biomechanically superior to monocortical placement and has been advocated for canine spinal surgery [3]. However, bicortical implant placement utilizing the vertebral body and pedicles in the canine cervical spine is fraught with potential complications due to narrow implantation corridors and their close proximity to critical neurovascular structures including the spinal cord, nerve roots, and vertebral artery [4,5,6,7,8,9]. Additional considerations for implant placement include varying dog sizes, breed, and individual anatomical variations and surgical positioning of the patient [10,11]. Strategies to mitigate this risk have included monocortical placement of implants in the vertebral body [5] as well as use of the vertebral transverse processes for implant placement [7], although such methods still require care to avoid compromise of neurovascular structures. Intraoperative guidance of implant placement has also been investigated, including the use of three-dimensionally (3D) printed guides (3DPG) [12,13,14,15,16,17].

Three-dimensional printing is a form of additive manufacturing in which images, such as those obtained with computed tomography (CT), are used to create patient-specific materials using computer-aided design (CAD) [18,19,20]. Various technologies exist to print a wide range of materials [18,19,20], and a number of studies describe the use of 3DPG for stabilization of the canine spine, with considerable variability in the processes used in 3DPG creation [11,12,13,14,15,16,17,21,22,23,24,25,26,27,28,29,30,31,32,33,34,35]. Many of these studies have not addressed 3DPG sterilizability and chronological feasibility of the printing process.

The objective of this study was to evaluate drill tracts created in cadaveric canine cervical vertebrae (C1-C7) using 3DPG made of an autoclavable surgical resin and produced using a novel printer which features rapid printing times as well as an automated post-print processing sequence. We hypothesized that these 3DPG would allow drill tracts to be accurately constrained to their preplanned trajectories and that there would be no significant difference between the drilling accuracy of an experienced surgeon and a novice surgeon.

## 2. Materials and Methods

### 2.1. Animals

Six mixed-breed canine cadavers (12–28 kg) were donated from a local shelter after humane euthanasia due to circumstances unrelated to the study. The cervical vertebral column and surrounding paraspinal musculature were isolated by disarticulating the atlanto-occipital joint and the C7-T1 connections. The vertebral segments were then wrapped in saline-soaked towels and frozen until imaging.

### 2.2. Computed Tomographic Imaging and 3DPG Design

The vertebral segments were thawed overnight at room temperature and imaged using a 16-slice helical CT scanner (SOMATOM Sensation; Siemens Medical Solutions, Malvern, PA, USA). Acquisition parameters were as follows: 1 mm slices with 0.7 mm overlap, kVp 120, mA 118, field of view 15.9 to 25.6 cm^2^, and B50S convolution kernel. These parameters were chosen because using thin slices with a significant overlap increases image accuracy [36,37,38] and because it closely matched clinical CT protocols typically used in our hospital. The vertebral segments were rewrapped in saline-soaked towels and refrozen.

**Figure 1:**
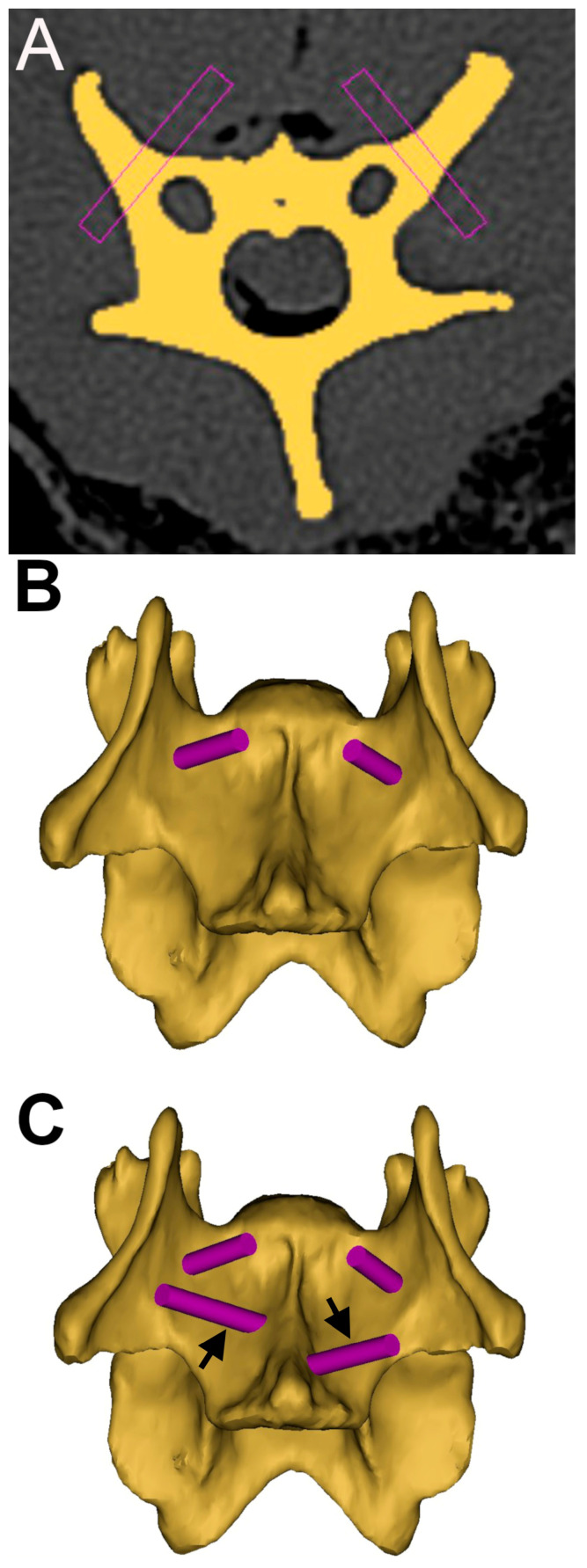
Screenshots from modeling software (Mimics) showing the following: (**A**) planned trajectories into the transverse processes of C4 (purple rectangles); (**B**) same trajectories shown as cylinders on a three-dimensional representation; (**C**) addition of two trajectories into the vertebral body (arrows).

The acquired images were imported into image processing and modeling software (Mimics, version 22.0; Materialise, Plymouth, MI, USA). Each cervical vertebra was segmented from the surrounding soft tissues and adjacent vertebrae, and a 3D model was created. Intended drill trajectories were planned using a cylinder function (Figure 1). We originally planned trajectories to simulate placement of a bicortically engaged, 3.5 mm cortical screw. However, implantation corridors between the vertebral canal and transverse foraminae were too narrow for most of the vertebrae to accept such an implant (or often even the 2.5 mm drill bit). Thus, we planned two monocortical trajectories within each vertebral body and two bicortical trajectories within the transverse processes of each vertebra, after the methods of Hettlich et al. [5] and Hicks et al. [7], with the exception of the atlas and axis. In each atlas, we planned two bicortical trajectories traversing the lateral masses. In the axis, we planned two monocortical trajectories into the vertebral body just caudal to the articulation with the atlas on either side and then a third monocortical trajectory in the caudal vertebral body just rostral to the endplate, after the method described by Platt et al. [39]. For each planned monocortical trajectory, we derived a depth using the “measure” function in the Mimics software, which served as the distance we could safely drill without breaching the vertebral or transverse foraminae.

**Figure 2:**
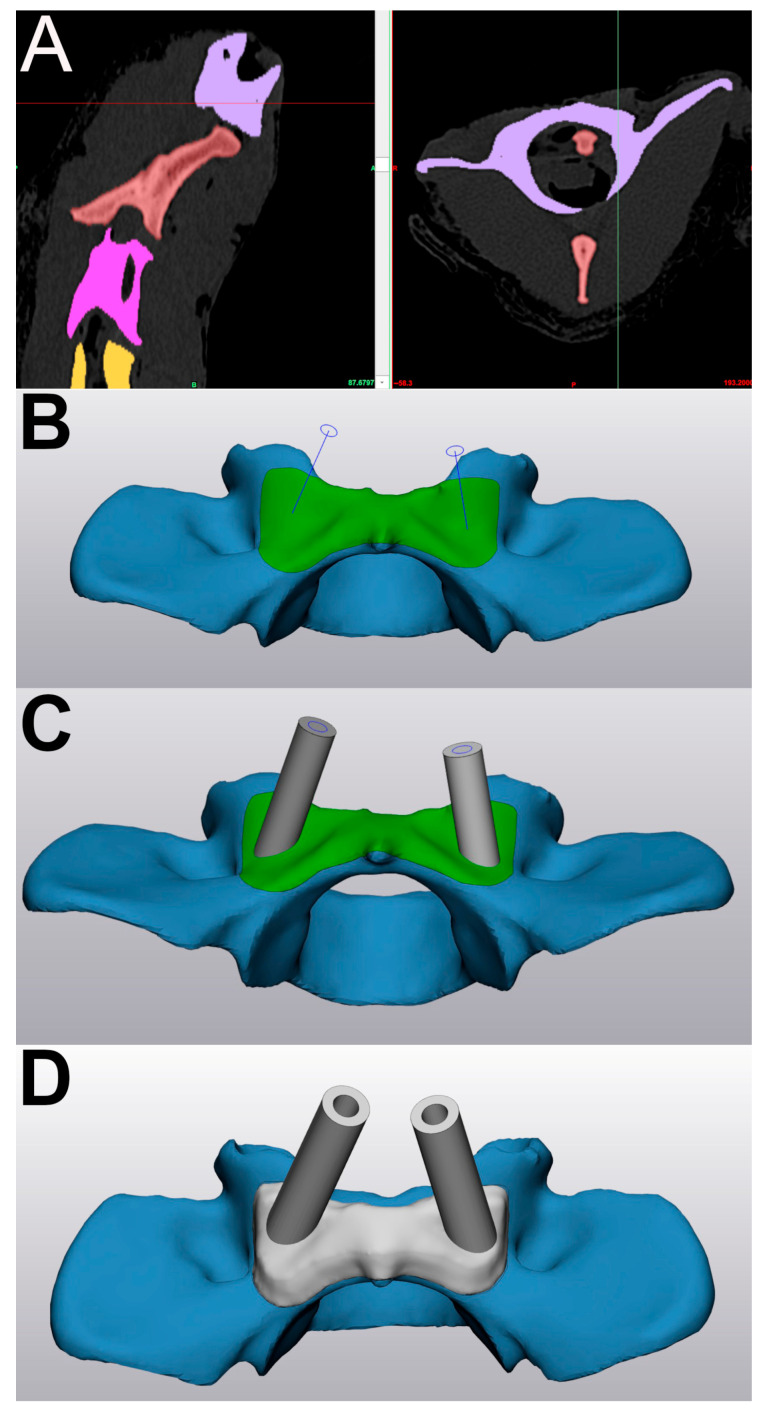
Screenshots of modeling (Mimics, (**A**)) and computer-aided design software (3-matic, (**B**–**D**)) showing the following: (**A**) Segmentation of cervical vertebrae, including atlas (light purple). (**B**) Model of atlas with base of drill guide contoured to ventral surface (green) and trajectories imported from Mimics (thin blue lines). (**C**) Trajectories represented as cylinders of appropriate drill width. (**D**) Fusion of cylinders with base to complete guide design.

The 3D objects were then imported into CAD software (3-matic, version 14; Materialise). The 3DPG were designed to fit a 2.5 mm steel drill sleeve (model 312.28; DePuy Synthes Vet, West Chester, PA, USA), intended for a 2.5 mm drill bit used to create a corridor to accommodate a 3.5 mm cortical screw. The drill bit and hypothetical implant sizes were chosen based on recommendations for dogs of this size [3]. The 3DPG were designed by first creating a 3 mm thick base contoured to fit the ventral surface of each vertebra, including portions of the medial surface of the transverse processes (Figure 2). Areas of the vertebral surface near anticipated hard-to-remove soft tissue (e.g., articular surfaces, disk spaces) were avoided in the creation of the base to minimize interference with 3DPG placement. Hollow cylinders were then oriented along the axis of the planned trajectories and merged with the base. The 3DPG were created for individual vertebrae and did not span intervertebral disk spaces. Several 3DPG were designed for each vertebra to avoid interference (crossing) between the guide cylinders on either side (typically one guide for the left side and one for the right). The 3DPG were labeled with their associated cadaver and vertebrae number, and the files were exported into 3D printing software (Magics, version 23.01; Materialise).

### 2.3. 3DPG and Biomodel Printing

The 3DPG printing was performed at the North Carolina State University Center for Additive Manufacturing and Logistics (CAMAL) using a 3D Printer (D50+, Rapid Shape GmbH, Heimsheim, Germany). The 3DPG were printed using Rapid Shape’s proprietary autoclavable dental resin. After printing, the 3DPG were washed with isopropyl alcohol in an automated machine (RS wash, Rapid Shape GmbH) and then cured using ultraviolet light (RS Cure, Rapid Shape GmbH). A biomodel of one cadaver spine (dog 1) was printed with a photocurable resin inkjet printer (Objet Connex 350; Stratasys, Eden Prairie, MN, USA) using a mixture of polymer (DM8425 Verowhite; Stratasys) and elastomer (TangoPlus; Stratasys), which mimics the tactile sensation of drilling bone [29,40].

### 2.4. Cadaver Surgeries

To evaluate the 3DPG fit along the ventral vertebral surface and the integrity of the 3DPG while drilling, we initially tested the 3DPG on the 3D biomodel (Supplemental Figure S1A). Accuracy was gauged by visual assessment and CT imaging of the biomodel. The cadaveric vertebral segments were thawed overnight and secured to a surgery table with bandaging tape in a position simulating dorsal recumbency. Muscle separation and retraction were performed in a manner simulating a live-animal ventral surgical approach (Supplemental Figure S1B). The 3DPG were held on the ventral vertebral surface, the drill sleeve was introduced into the guide cylinders, and an electric surgical drill (DePuy Synthes Vet, West Chester, PA, USA) with a 2.5 mm drill bit was used to create the drill tracts. A silicone drill stop was used to constrain depth when creating monocortical drill tracts. To assess the effect of surgical experience on drilling accuracy, drill tracts were randomly assigned to a surgeon with >25 years of experience (C.L.M.) or an inexperienced surgeon (second-year veterinary student, C.J.P.). Finally, repeat CT imaging was performed on the cervical segments using an identical protocol to the presurgical scans.

### 2.5. Assessment of Drill Tract Accuracy

**Figure 3:**
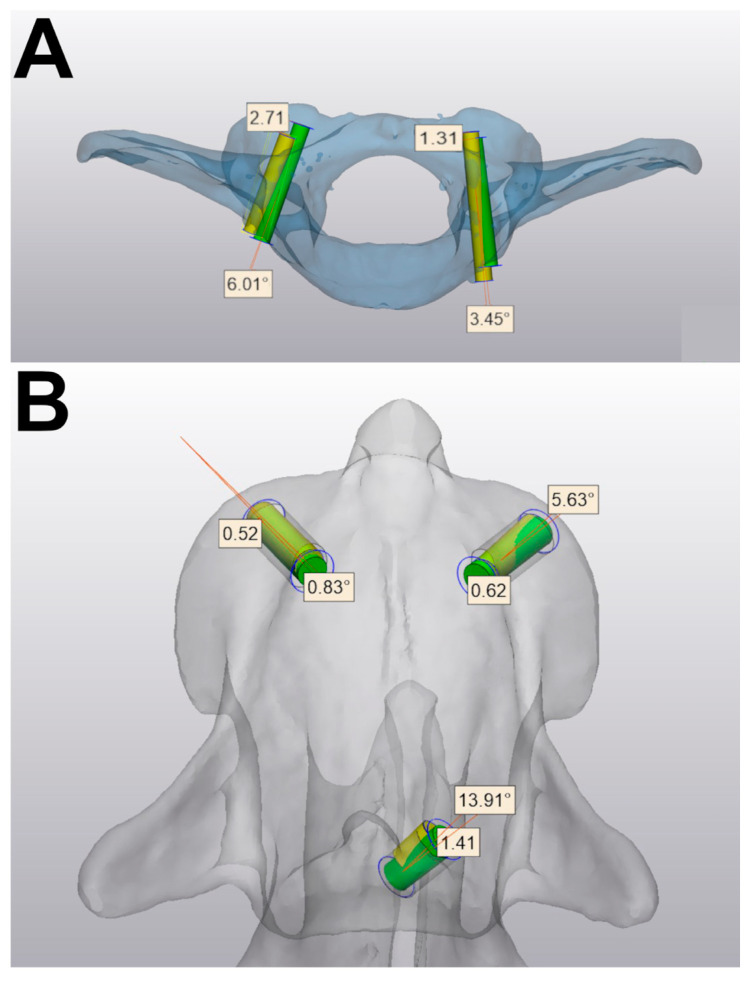
Comparison of intended drill trajectory (green) and actual drill tract (yellow) in a representative atlas (**A**) and axis (**B**). The angular and entry point deviations are shown for each pair of cylinders.

The post-operative CT images were imported into Mimics and segmented in the same fashion as the preoperative images. A 3D image of each post-operative vertebra was created and aligned with the corresponding preoperative 3D image, and measurement functions (Create Analytical Cylinder, WaveBrush Marker) were used to outline the drill tracts and compare them with intended trajectories (Figure 3 and Supplemental Figure S2). Accuracy was assessed by measuring deviation from the intended entry point (EPD) for each tract as a linear measurement from the center of each cylinder and angular deviation (AD) of the cylinder centers. These errors were recorded as deviations in three planes (x, y, z) generated within the modeling software. Unintended cortical bone breaches were also recorded.

### 2.6. Statistical Analysis

Descriptive statistics were produced for EPD and AD. D’Agostino and Pearson normality testing showed that these values were not normally distributed, and nonparametric methods were used to compare groups. A Mann–Whitney test was used to compare values between the inexperienced and experienced surgeons and between vertebral location (vertebral body versus transverse process). Comparisons between different vertebrae were performed using Kruskal–Wallis tests with Dunn’s multiple comparison testing. For all analyses, *p* values < 0.05 were considered significant. Statistical analyses were conducted using commercial software (Prism version 9.0.0, GraphPad Software, La Jolla, CA, USA).

## 3. Results

We planned to create 150 drill tracts (25 tracts/dog; 2/atlas, 3/axis, 4 each for C3–7) using 84 3DPG. However, four of the 3DPG had errors during printing or broke during introduction of the drill sleeve and were not used in the cadaver surgeries, which affected six drill tracts (Dog 3, C6 transverse process and vertebral body; Dog 6, C3 transverse process and vertebral body, C5 vertebral body, and C6 transverse process). Therefore, a total of 144 drill tracts were created in 42 vertebrae, with the novice surgeon creating 70 tracts and the experienced surgeon creating 74 tracts. The 3DPG subjectively conformed very well to the surface of the 3D printed biomodel, and we successfully created drill tracts without any unintended cortical breaches. The metal drill sleeve fit snugly into all printed 3DPG, although the sleeve was double-sided and generated some torque on the lighter 3DPG that had to be accounted for while drilling.

**Figure 4:**
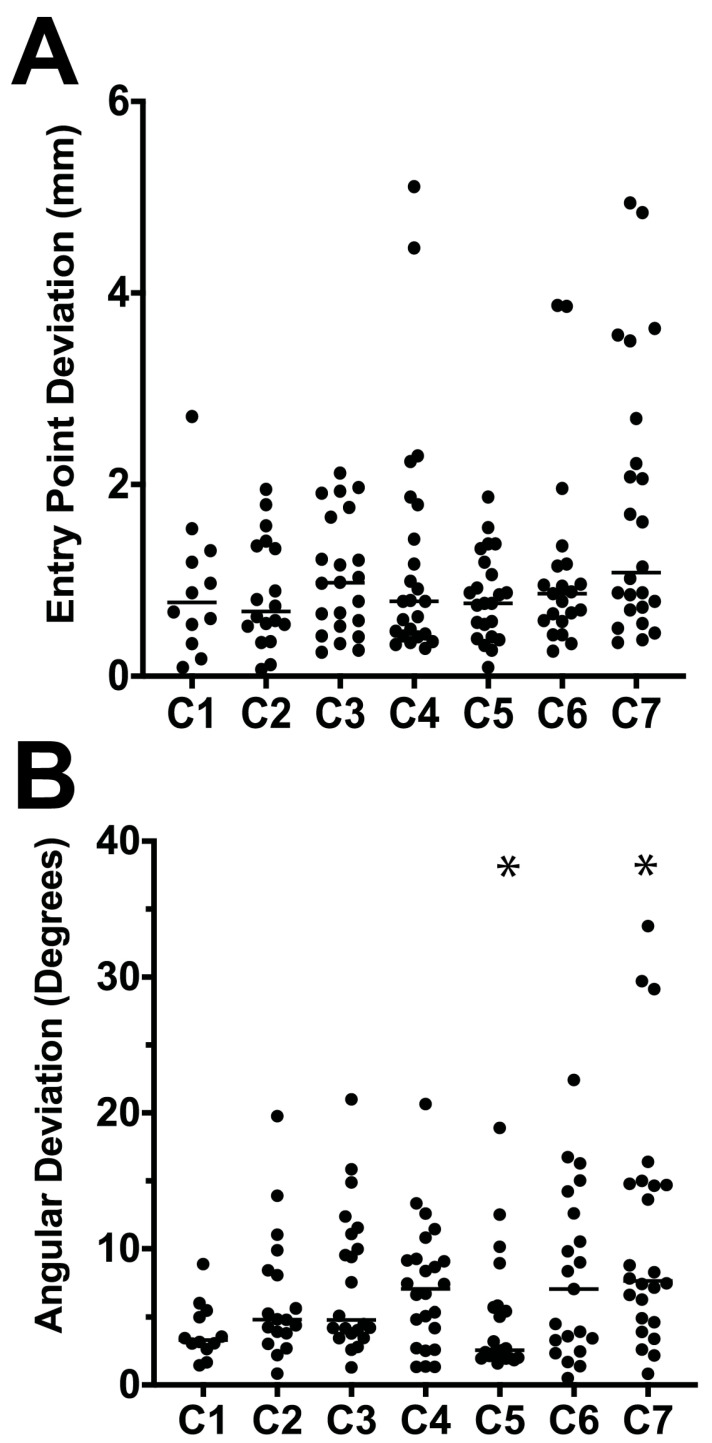
Scatter plots showing (**A**) entry point deviations (mm) and (**B**) angular deviations (°) from intended drill tract trajectories by vertebra for all five cadaveric spines. Medians are shown as horizontal lines. Angular deviations were significantly larger for C7 than for C5 (* *p* = 0.01).

The use of 3DPG in cadavers was similar, with subjectively accurate fitting of the guides to their intended vertebral surfaces. The overall mean EPD was 1.1 mm (median 0.9 mm, range 0.1–5.1 mm) while the overall mean AD was 7.3° (median 5.2°, range 0.5–33.8°). There was no difference in overall EPD (*p* = 0.85) or AD (*p* = 0.20) between the inexperienced and experienced surgeons. The EPD and AD for each vertebra by surgeon are shown in Table 1. When comparing vertebrae, AD was larger for C7 than C5 (*p* = 0.01), but EPD amongst vertebrae did not differ statistically (*p* = 0.30, Figure 4). When comparing vertebral location, AD was larger for drill tracts made in transverse processes than vertebral bodies (*p* = 0.007), but there were no differences in EPD between these locations (*p* = 0.54). There were no unintended cortical bone breaches.

## 4. Discussion

During this study, 80 custom designed 3DPG were used to create 144 drill tracts in the cervical vertebrae of six canine cadavers. The 3DPG conformed well to their intended vertebral surfaces. Most drill tracts produced had minimal errors when considering EPD and AD and all were considered clinically acceptable, without unintended cortical breaches. There were no differences in EPD or AD when comparing tracts made by a novice surgeon versus an experienced surgeon.

Canine cervical vertebrae are affected by various conditions that often require surgical stabilization, including cervical spondylomyelopathy, atlanto-axial subluxation, and trauma, and the placement of pins or screws with polymethylmethacrylate is a commonly used technique [1,2,3,10,41,42,43]. Biomechanical studies have shown that bicortical pins with polymethylmethacrylate result in significantly increased stiffness and resistance to intervertebral motion compared with non-instrumented cervical spinal segments [7,8], and bicortical implant placement has been advocated as ideal for the canine spine [3]. However, this implant strategy is technically challenging because of a consistently narrow implant corridor and is fraught with potential complications including damage to the spinal cord, vertebral artery, or nerve roots. Insertion of implants starting on the ventral midline at an angle of 30–35° from the vertical has been suggested to avoid critical neurovascular structures [8,43] but results in violation of the vertebral foramen, transverse foraminae or intervertebral foraminae in a large proportion of dogs [4,5,7,8]. A morphometric study that assessed implantation corridors in vertebral bodies of 86 C2–C6 vertebrae found that 68.6% measured less than 2.5 mm in dogs with an average weight of 22 kg [9]. We similarly found planning safe trajectories for bicortical drill insertion of a 2.5 mm drill bit impossible in most vertebrae for dogs weighing 12–28 kg in this study. Although we could have reduced our drill bit size to 1.5 mm (to accommodate a 2.0 mm screw), we judged this screw size to be too small for most dogs in this study, risking screw breakage if this strategy was used in a clinical scenario. Since a monocortical implant approach for cervical spine stabilization has been found to be biomechanically comparable to bicortical fixation [5] and is commonly used in veterinary neurosurgery, we designed 3DPG to simulate this strategy as well as an alternate approach using transverse processes [7].

The 3DPG in our study created clinically acceptable drill tracts with minimal EPD (median 0.9 mm) and AD (median 5.2°) and without unintended vertebral cortical violations. Several factors may have contributed to these trajectory errors. Factors contributing to CT image quality and subsequent accuracy of 3D printed objects include the amount of radiation applied to the tissue (kVp, mA), field of view, slice thickness, and slice overlap. Generally, higher kVp and mA improve image resolution and print accuracy, with kVp potentially having a greater impact [44,45]. However, errors can occur in the z-plane (typically the craniocaudal axis when imaging the vertebral column) due to partial volume averaging at the edges of a slice. To improve image resolution, thinner slices and overlapping slices (pitch reduction) can be used [36,37,38]. We used 1 mm slices with a 0.7 mm overlap based on these considerations, which also aligns with clinical imaging protocols used in our hospital.

The 3DPG were designed to maximize contact with the ventral surface of the vertebral bodies to improve conformation and provide stability during drilling. However, soft tissue remnants may have interfered with this conformation. The 3DPG surfaces that contact the vertebrae were digitally smoothed in the design phase, which could have resulted in some inaccuracies. Larger angular deviations were observed for the C7 vertebra and may have been related to the increased mobility of this vertebra due to its position at the end of the disarticulated segment. Drill bit skiving on the surface of the vertebrae was evident at times and contributed to EPD for some trajectories. The use of non-skiving drill bits may help to reduce this error and is worthy of future exploration. We designed the 3DPG to accommodate a standard double-sided metal drill sleeve, which protected the guides from the drill bit but was much heavier than the 3DPG themselves. This complicated the technical aspects of the procedure, as both the 3DPG and sleeve had to be held in place and stabilized during drilling and likely contributed to trajectory errors. A customized drill sleeve will likely improve upon this technique. Finally, there is some error intrinsic in the method that we used to assess EPD and AD, which required overlay of the preoperative and post-operative CT images. As the images were not captured at identical slice locations, this led to a small amount of error inherent in image alignment. We attempted to estimate this error by performing 10 successive overlays for each vertebra in one cadaver and quantifying the mean distance error relative to the width of the vertebra in question. Using this method, we estimate that the mean distance error associated with this step was approximately 0.6–0.9%.

The results of studies in humans provide evidence that the use of 3DPG can improve the accuracy of spinal implant placement compared with freehand techniques, and this improvement is dramatic in some reports [46,47,48,49,50,51,52]. Additional benefits of 3DPG compared with traditional methods noted in some studies include shorter operative times [47,49,50,51], reduced blood loss [47,49,51], and decreased use of fluoroscopy during the procedure [46,47,49].

A number of studies have now demonstrated the feasibility of using 3DPG in different regions of the canine spine including the cervical [12,14,15,16,17,34], thoracolumbar [11,23,24,25,26,27,28,29,31,35] and lumbosacral segments [21,32,33]. Our study is the only one that we are aware of to evaluate the entire cervical spine, as others focused on more specific regions, including C1–2 [14,15,16,17], C4–7 [12] and C5–6 [34].

There are various ways to assess the utility of 3DPG. While some studies have attempted to rigorously quantify drill tract or implant placement accuracy by measuring EPD (and exit point deviation) [17,24,33], AD [16,21,25,28,34] or both [13,26,29], others have used a modified Zdichavsky score [11,15,16,23,25,26,27,28,29] or have adapted other human scales quantifying the degree of unintended cortical breeches using various length increments or proportions of screw diameter [12,13,22,24,32,33]. Still others have used simple visual inspection of post-operative implant placement [14,21,31,35]. We chose to assess EPD and AD to thoroughly define any errors made in drill tract trajectories to better compare surgeons of different experiences and to lay the groundwork for future improvements in the technique. The mean EPD (1.1 mm) and AD (7.3°) in our study is similar to other studies utilizing 3DPG for canine vertebrae, with mean values ranging from 0.51 to 2.43 mm [13,17,24,26,29,33] and 2.03 to 6.5° [13,25,26,28,29,34], respectively. We recorded cortical bone breaches as a clinically relevant measure of safety and our study also performed favorably with this assessment, with no cortical breaches created. Regardless of assessment technique, the consensus from these studies is that use of 3DPG facilitates safe and accurate drilling and implant placement within canine vertebrae.

Several studies have compared the accuracy of using 3DPG for creating drill tracts or placing implants in canine vertebrae versus freehand drilling techniques [16,22,27,30,35]. Most have shown improved accuracy through assessment of a modified Zdichavsky grade [16,27,30]. Shin et al. compared Kirschner wire placement into T12-L1 of 21 small breed cadavers by an experienced surgeon using a freehand technique versus seven novice surgeons using 3DPG and found that the novices placed 63/63 (100%) screws completely within bone versus 61/63 (97%) for the experienced surgeon. The post-operative angles of the K-wires were also significantly closer to the intended trajectory angles in the novice group [35]. Bongers et al. compared an experienced versus novice surgeon placing L7-S1 implants and found no differences between them for either freehand or 3DPG conditions [22].

In our study, we found no significant differences in linear or angular deviations when comparing the novice and experienced surgeons, and no drill tracts violated cortical bone. Together with other studies, this demonstrates the effectiveness of 3DPG in constraining drill tracts to intended trajectories and improving outcomes, independent of surgical expertise [28,29,35]. However, in some other studies, the use of 3DPG either did not improve implant placement [22] or did but relatively underperformed compared to other studies [27]. The reasons for these discrepancies are not entirely clear but may be related to the shorter length of the 3DPG cylinder designed to accommodate the drill bit or sleeve. In preliminary studies of the cervical spine, we found that 3DPG with shorter cylinders had reduced accuracy and more violations of the vertebral and transverse foraminae. Other benefits of using 3DPG demonstrated in human studies (e.g., reduced blood loss) remain to be investigated, although at least one cadaveric canine study has shown a reduced time for implant placement with 3DPG compared with a freehand technique [30].

Additive manufacturing continues to revolutionize the medical field with rapidly expanding printing technologies and materials. Many of these techniques have been pioneered in the dental and orthodontic space [20,53]. Sterilization of some materials presents challenges, particularly plastics such as Polylactic Acid (PLA), which cannot tolerate the high temperatures associated with steam sterilization. Low-temperature sterilization with ethylene oxide has been used in several veterinary studies for 3DPG made from acrylic or PLA polymers [14,17,24,33], although this is increasingly difficult to perform due to human health concerns [54,55]. The use of other low-temperature sterilization techniques, such as hydrogen peroxide, ozone, and ionizing or UV radiation, and their effects on 3DPG integrity is an area worthy of future investigation [56,57].

To address this challenge, we used a print system that employs a proprietary autoclavable surgical resin and a rapid print speed with automated post processing, which allowed the creation of 3DPG in a short amount of time (~1 h). Several other veterinary studies have used systems that employ autoclavable resins, although the print times for such systems are typically much longer [12,23,26]. Shortening the printing process is an important step in successfully adapting these technologies for patients requiring rapid surgical interventions (e.g., traumatic spinal fractures and luxations). Several of our 3DPG broke during drill sleeve placement in the guide cylinder, typically at the junctions of the cylinders and the guide base, suggesting that this transition point was inherently weak or that torque from the heavier steel drill sleeve overwhelmed the composite resistance at these junctions. This might be avoided by increasing the thickness of the 3DPG base, reinforcing the cylinder–base junction, or by using a custom-designed drill sleeve with a single arm to reduce torque during drilling. Although our 3DPG were suitable to be autoclaved, our study did not assess the potential effect that the sterilization process would have on the integrity and accuracy of the guides, which may warrant further investigation.

Our study has several limitations. We did not place implants into the vertebrae after drilling a 2.5 mm pilot hole. Thus, insertion of the appropriate 3.5 mm screw might have created unintentional cortical breaches. However, implant placement would have only extended the corridor by 0.5 mm on each side, a distance that is unlikely to result in clinically relevant complications. Additionally, our study was limited by the use of cadaveric spines instead of live animals, with only a simulated ventral surgical approach. This eliminated anatomic structures such as the trachea, esophagus, carotid arteries, xiphoid, and caudal cervical musculature, which could have made drill trajectories more difficult. The presence of hemorrhage was also eliminated, which could complicate the surgical approach, including the meticulous removal of soft tissues over the vertebrae’s ventral surfaces and transverse processes that is critical to correct 3DPG placement.

## 5. Conclusions

In conclusion, the 3DPG used in this study allowed the creation of drill tracts with small amounts of EPD and AD from the intended path and without cortical bone violations. The guides were developed by an individual who had recently acquired knowledge in 3D printing, and this technique was easily used by both an experienced and a novice surgeon, who displayed similar drill tract accuracies. The novel printing technology used in this study resulted in rapid printing times and post-printing processes to create autoclave-ready 3DPG that would be immediately applicable to clinical cases.

## Figures and Tables

**Table 1:** Entry point (mm) and angular (°) drill tract deviations from planned trajectories for each vertebra by surgeon.

	C1	C2	C3	C4	C5	C6	C7
**EPD** **(All)**	0.9 ± 0.7 0.8 (0.1–2.7)	0.9 ± 0.6 0.7 (0.1–2.0)	1.0 ± 0.6 1.0 (0.3–2.1)	1.2 ± 1.3 0.8 (0.3–5.1)	0.8 ± 0.5 0.8 (0.1–1.9)	1.1 ± 1.0 0.9 (0.3–3.9)	1.8 ± 1.4 1.1 (0.4–4.9)
**AD** **(All)**	3.9 ± 2.1 3.3 (1.4–8.9)	6.5 ± 4.7 4.8 (0.8–19.8)	7.6 ± 5.2 4.8 (1.3–21.0)	7.2 ± 4.6 7.1 (1.3–20.7)	4.8 ± 4.3 ^a^ 2.6 (1.6–18.9)	8.1 ± 6.2 7.0 (0.5–22.4)	11.0 ± 8.9 ^a^ 7.6 (0.8–33.8)
**EPD** **(E)**	1.4 ± 0.8 1.3 (0.6–2.7)	0.7 ± 0.5 0.6 (0.1–1.8)	1.0 ± 0.6 0.8 (0.3–2.0)	1.6 ± 1.6 0.8 (0.4–5.1)	0.9 ± 0.5 0.8 (0.3–1.9)	1.2 ± 1.0 1.1 (0.3–3.9)	1.7 ± 1.7 0.9 (0.4–4.9)
**AD** **(E)**	4.6 ± 1.3 5.0 (3.2–6.0)	6.8 ± 5.3 4.6 (2.2–19.8)	8.6 ± 4.8 9.5 (1.3–15.9)	8.7 ± 5.1 8.4 (1.4–20.7)	4.9 ± 5.0 2.4 (1.9–18.9)	7.8 ± 6.6 6.5 (0.5–22.4)	10.3 ± 8.0 7.5 (0.8–29.7)
**EPD** **(N)**	0.6 ± 0.4 0.5 (0.1–1.2)	1.1 ± 0.6 1.1 (0.5–2.0)	1.1 ± 0.6 1.0 (0.3–2.1)	0.8 ± 0.5 0.8 (0.3–1.9)	0.7 ± 0.4 0.9 (0.1–1.3)	1.0 ± 1.0 0.8 (0.3–3.9)	1.8 ± 1.2 1.6 (0.5–3.6)
**AD** **(N)**	3.5 ± 2.5 3.1 (1.4–8.9)	5.8 ± 3.6 6.4 (0.8–9.9)	6.5 ± 5.5 4.2 (2.6–21.0)	5.4 ± 3.2 4.8 (1.3–9.3)	4.6 ± 3.6 2.7 (1.9–12.5)	8.3 ± 6.2 7.0 (1.4–16.8)	11.6 ± 10.0 8.3 (2.2–33.8)

Abbreviations: AD, angular deviation; EPD, entry point deviation; E, experienced surgeon; N, novice surgeon. Data presented are mean ± SD and median (range) for six dogs. ^a^ Significantly different (*p* < 0.05).

## Data Availability

The original contributions presented in this study are included in the article/Supplementary Material. Further inquiries can be directed to the corresponding authors.

## Supplementary Materials

The following supporting material can be downloaded at https://www.mdpi.com/article/10.3390/vetsci12121190/s1, Supplementary Figure S1, 3D printed drill guides in place on a model spine and on a cadaveric spine; Supplementary Figure S2, Workflow for analysis of drill tract accuracy.

## Abbreviations

The following abbreviations are used in this manuscript:

3D	Three-dimensionally
3DPG	Three-dimensionally printed drill guides
AD	Angular deviation
CAD	Computer-aided design
EPD	Entry point deviation
PLA	Polylactic acid
