# Supplementary material for: Accuracy of Three-Dimensionally Printed, Patient-Specific Drill Guides for Implant Placement in Canine Cervical Vertebrae: A Cadaveric Study
Source: Vet Sci. 2025 Dec 12;12(12):1190. doi: 10.3390/vetsci12121190 (PMC12737624; doi:10.3390/vetsci12121190)
Supplement: Supplementary file 1 [file vetsci-12-01190-s001.zip › vetsci-3927116-supplementary.pdf]

Supplementary Files for "Accuracy of three-dimensionally printed, patient-specific drill guides for implant placement in canine cervical vertebrae: a cadaveric study"

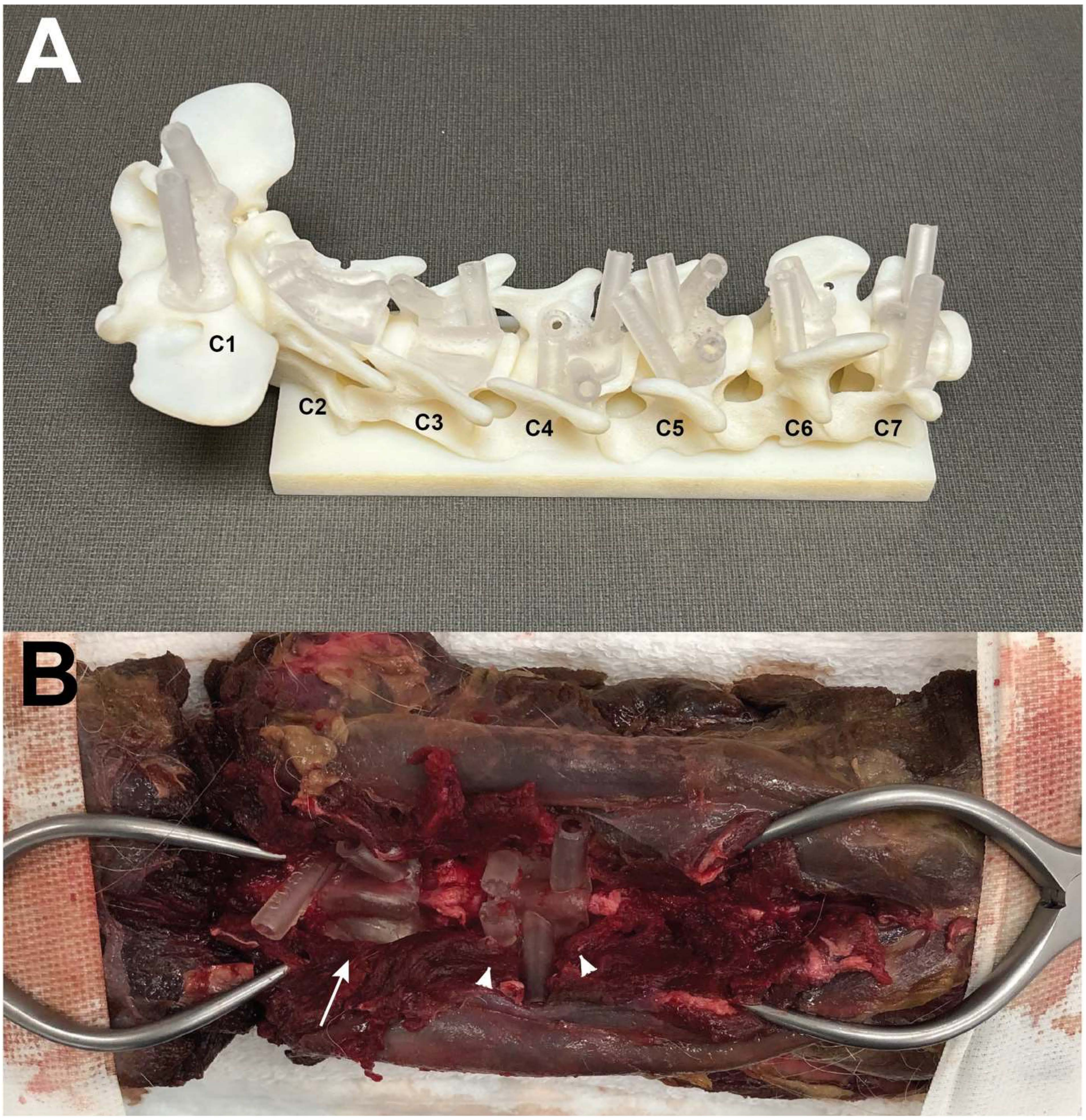

Supplemental Figure S1. Image showing (A) 3DPG placed on biomodel. Note that the guides are specific to each vertebra and most (C1, C2, C3, C6, C7) have cylinders associated with 2 trajectories while C4 and C5 have 4 trajectory cylinders on one guide. (B) The 3DPG for C3 (white arrow) and C4 (white arrowheads) in place on a cadaveric spine prior to drill tract creation.

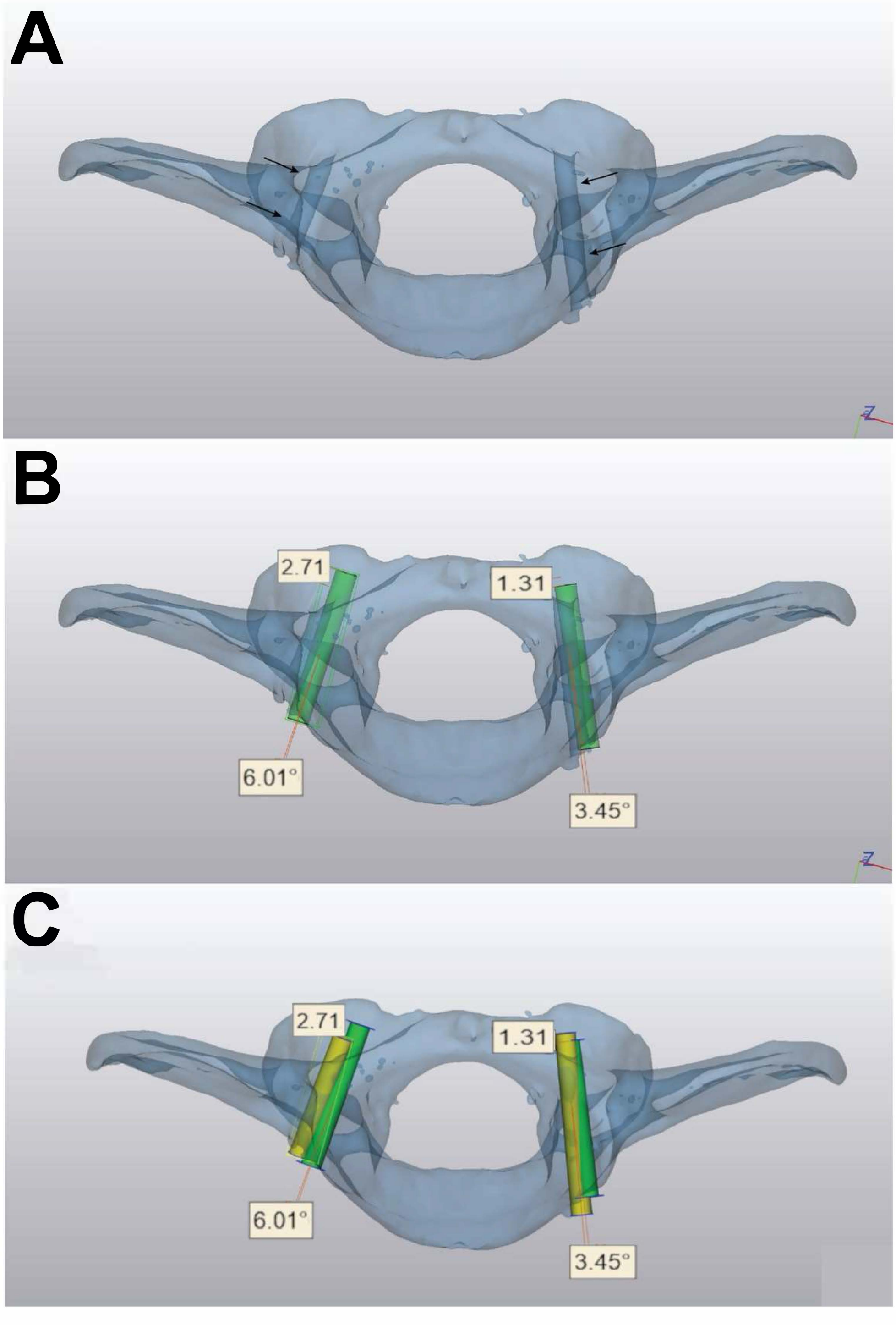

Supplemental Figure S2. Screenshots showing workflow for analysis of drill tract accuracy. (A) 3D rendering of an atlas from a cadaveric spine after drill tract creation (black arrows). (B) 3D rendered atlas from (A) with intended drill tract trajectories (green cylinders) overlain. (C) Image from (B) with actual drill tracts highlighted (yellow cylinder). The measured angular and entry point deviations are shown for each pair of cylinders in (B) and (C).
